# Characterization and fine mapping of a novel barley Stage Green-Revertible Albino Gene (*HvSGRA*) by Bulked Segregant Analysis based on SSR assay and Specific Length Amplified Fragment Sequencing

**DOI:** 10.1186/s12864-015-2015-1

**Published:** 2015-10-23

**Authors:** Dandan Qin, Jing Dong, Fuchao Xu, Ganggang Guo, Shuangtao Ge, Qing Xu, Yuxin Xu, Meifang Li

**Affiliations:** Institute of food crops, Hubei Academy of Agricultural Science, Hubei Wuhan, 430064 China; Hubei Key Laboratory of Food Crop Germplasm and Genetic Improvement, Hubei Wuhan, 430064 China; Institute of Crop Science, Chinese Academy of Agricultural Sciences, Beijing, 100081 China; NO.1 Middle School Affiliated to Central China Normal University, Hubei Wuhan, 430223 China

**Keywords:** Fine mapping, Barley, Stage Green-Revertible Albino, Specific Length Amplified Fragment Sequencing, Bulked Segregant Analysis

## Abstract

**Background:**

Leaf color variations are common in plants. Herein we describe a natural mutant of barley cultivar Edamai No.6, *whs18*, whose leaf color showed stable and inheritable stage-green-revertible-albino under field condition.

**Methods:**

Bulked Segregant Analysis (BSA) based on SSR assay and Specific Length Amplified Fragment Sequencing (SLAF-seq) was used to map the candidate gene for this trait.

**Results:**

We found that leaf color of *whs18* was green at seedling stage, while the seventh or eighth leaf began to show etiolation, and albino leaves emerged after a short period. The newly emerged leaves began to show stripe white before jointing stage, and normal green leaves emerged gradually. The duration of *whs18* with abnormal leaf color lasted for about 3 months, which had some negative impacts on yield-related-traits. Further investigations showed that the variation was associated with changes in chlorophyII content and chloroplast development. Genetic analysis revealed that the trait was controlled by a single recessive nuclear gene, and was designed as *HvSGRA* in this study. Based on the F_2_ population derived from Edamai No.9706 and *whs18*, we initially mapped the *HvSGRA* gene on the short arm of chromosome 2H using SSR and BSA. *GBMS247* on 2HS showed co-segregation with *HvSGRA*. The genetic distance between the other marker *GBM1187* and *HvSGRA* was 1.2 cM. Further analysis using BSA with SLAF-seq also identified this region as candidate region. Finally, *HvSGRA* interval was narrowed to 0.4 cM between morex_contig_160447 and morex_contig_92239, which were anchored to two adjacent FP contigs, contig_34437 and contig_46434, respectively. Furthermore, six putative genes with high-confidence in this interval were identified by POPSEQ. Further analysis showed that the substitution from C to A in the third exon of fructokinase-1-like gene generated a premature stop codon in *whs18*, which may lead to loss function of this gene.

**Conclusions:**

Using SSR and SLAF-seq in conjunction with BSA, we mapped *HvSGRA* within two adjacent FP contigs of barley. The mutation of fructokinase-1-like gene in *whs18* may cause the stage green-revertible albino of barley. The current study lays foundation for hierarchical map-based cloning of *HvSGRA* and utilizing the gene/trait as a visualized maker in molecular breeding in future.

**Electronic supplementary material:**

The online version of this article (doi:10.1186/s12864-015-2015-1) contains supplementary material, which is available to authorized users.

## Background

Variations in leaf colors are very special in the kingdom of plants, and different kinds of them have been reported [1]. Thus far, more than 80 Chl-deficient mutants have been discovered in rice, and they are referred to as *virescent*, *stripe*, *albino*, *chlorina*, *zebra* and *yellow variegated* according to their diverse phenotypes (http://www.shigen.nig.ac.jp/rice/oryzabase/) [2]. Leaf color mutants have been utilized extensively in theoretical studies during the last several years. For example, leaf color mutants have been used to interpret the development of chloroplasts and metabolism of chlorophyll in plants [3, 4], and to illustrate the pathways of photosynthesis [5], the mechanisms of photomorphism [6], metabolism of phytohormones [7] and molecular mechanisms of disease resistance [8].

Green-revertible albino is a special type of leaf color variation. Leaf color of this type of mutant is albino under certain conditions or at certain developmental stages, while it shows green leaves gradually and thus guarantees the mutant to be able to grow and mature normally [9], such as the rice mutant *Qiufeng M* [9] and *low temperature albino 1* [10]. As a visualized marker, this type of variation has been used successfully in breeding male-sterile lines in rice and enhancing the seed purify of hybrids in plants [11–14]. Furthermore, some of these type of leaf color variations are reported to be controlled by temperature, such as the rice mutant *low temperature albino 1* [10] and maize inbred line A661 [15]. They can also be used to illustrate the mechanisms of sense and response of temperature in plant.

The completion of draft genome sequences of several higher plant species has opened an unprecedented opportunity for functional genomics studies in them [14, 16]. In rice, more than 40 green-revertible albino mutants have been identified [1]. Several genes responsible for this type of mutation have also been identified using the strategy of forward genetics. These genes are involved in diverse physiological processes. For example, one base substitution of C to T in the coding region of chloroplast protein synthesis elongation factor Tu could result in the green-revertible albino [17]. The silence of cytochrome P450 gene *CYP-2* maybe responsible for the low temperature induced seedling-specific albino [18]. The 5-bp deletion in the coding region of the pentatricopeptide repeat (PPR) gene generated a premature stop codon in rice mutant *young seedling albino* and caused the seedling-specific albino phenotype [14]. The 45-bp insertion occurred in the first exon of a heme oxygenase gene may lead to the green-yellow phenotype in rice [19]. In maize, “zebra7 (*zb7*)” showed transverse green/yellow striped leaves in young plants, and map-based-cloning demonstrated that zb7 encoded the lspH protein with a mis-sense mutation in a conserved region [16].

On the other hand, combining classical bulked segregant analysis (BSA) with Next Generation Sequencing (NGS) technology has made gene cloning essentially a single-step computational procedure once a mapping population has been established [20]. Recently developed Specific Length Amplified Fragment Sequencing (SLAF-seq) is an efficient method of large-scale genotyping, which is based on reduced representation library (RRL) [21] and high-throughput sequencing [22]. SLAF-seq has been widely employed in many species [23–31], and was proven to be an effective strategy for construction of high density genetic map [23–27, 29], identification of gene for qualitative trait [28, 30] as well as QTLs for quantitative traits [24, 26, 31].

As the fourth cereal crop of the world, barley (*Hordeum vulgare* L., 2n = 2H = 14) provides economically important source of human and animal nutrition and underpins the malting and brewing industries. Barley also performs as a genetic model species for Triticeae genomics [32]. The recently released whole genome sequence of barley cv. Morex [33] has opened an unprecedented opportunity for performing functional genomics studies in barley. However, the genetic basis of leaf color variations in barley was largely unexplored.

In the present study, traditional SSR marker assays and SLAF-seq in conjunction with BSA were utilized to fine map a novel stage green-revertible albino gene (*HvSGRA*) in barley mutant *whs18*. The mutant was originally isolated from the malting barley variety Edamai No. 6 and showed stable and inheritable character of stage green-revertible albino. The candidate genes were also discussed and analyzed in this study. The current study will provide a basis to clone and utilizing the gene that is responsible for the albino trait in the future.

## Results

### Phenotypic characterization of the barley stage green-revertible albino mutant “*whs18*”

**Fig. 1:**
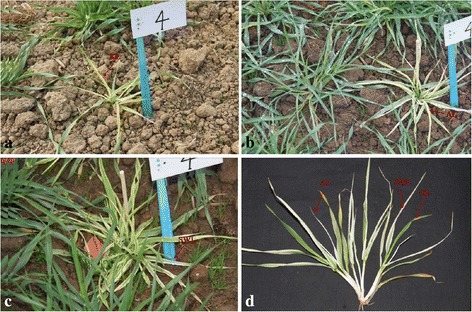
The dynamic leaf color variation of *whs18* under field condition. **a** Etiolated leaf **b** Albino leaf **c** Strip white leaf **d** Stereo leaf color performance

“*whs18*” was a natural mutant of the malting barley variety “Edamai No. 6”. After three years’ observation, we found that leaf color of *whs18* showed stage green-revertible albino under the field condition. To be detailed, *whs18* had wild-type leaf color at the seedling stage, while the seventh or eighth and later leaves showed etiolation (Fig. 1a), and albino leaves (Fig. 1b) emerged soon. The newly emerged leaves began to show stripe white (Fig. 1b, c) before the jointing stage, and normal green leaves (Fig. 1c) emerged gradually before heading time. Thus, leaf color of *whs18* could harbor the stereo model of “albino-stripe white-green” (Fig. 1d) after green leaves appeared again. Leaves with different color at the corresponding stage were called green leaf at the seedling stage (GL), etiolated leaf (EL), albinistic leaf (AL), stripe white (SW), respectively. The whole developmental period with abnormal leaf color generally lasted for about 3 months.

### The impact of temperature on the stage green-revertible albino of *whs18*

**Fig. 2:**
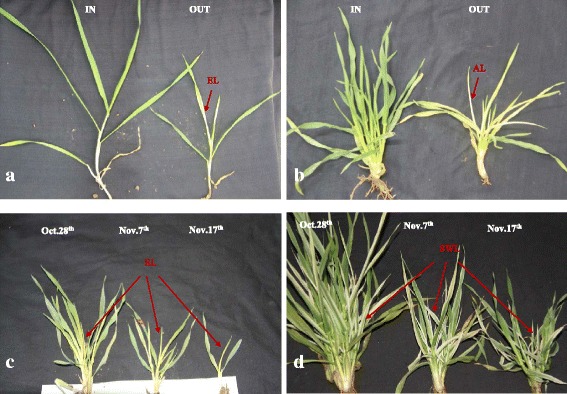
The phenotype of *whs18* under different temperature treatment. **a** T1: *whs18* grown inside or outside of incubator **b** T2: *whs18* grown inside or outside of plastic membrane **c** T3-1: *whs18* sowed at different date **d** T3-2: *whs18* sowed at different date

To analyze whether the color variation of *whs18* was induced by environment, three different temperature treatments (See methods) were conducted on *whs18*. After one month’s temperature treatment, we found that both of the seedlings in the incubator (Fig. 2a, left) and under the plastic membrane (Fig. 2b, left) grew much faster than the seedlings grown under normal condition (Fig. 2a and b, right) due to the warmer temperature. Meanwhile, the seedlings grown outside began to show ELs, and showed stage green-revertible albino as expected (Fig. 2a and b, right). However, we didn’t observe any color variation for the seedlings either in the incubator or under the plastic membrane (Fig. 2a and b, left). Another analysis showed that when encountered a period of low temperature, ELs and ALs emerged in all of the seedlings planted at three different dates (Fig. 2c), even though they have developed quite different numbers of leaves (Fig. 2c). The newly emerged leaves also showed green nearly at the same time (Fig. 2d).

### Effect of stage green-revertible albino on agronomic traits

To investigate the effect of the stage albino on agronomic trait of barley, we measured some yield related traits in Edamai No.6 and *whs18*. As shown in Table 1, heading date (HD) of *whs18* was 8 days’ later than Edamai No.6. Furthermore, stage albino significantly reduced the plant height (PL) and length of the main spike (LS) of barley for about 10 cm and 0.8 cm, respectively. Spike number per plant (SNP) and weight of 1000 grains (WTG) were also decreased significantly by stage albino. While it seemed that the stage green-revertible albino didn’t have any significant effect on grain number of the main spike (GNS).

**Table 1:** Comparison of agronomic traits between Edamai No. 6 and *whs18* (*p* = 0.05)

	HD	PL(cm)	SNP	LS(cm)	GNS	WTG(g)
Edamai No. 6	Mar.21	77.4	6.7	7.1	33.3	43.6
*whs18*	Mar.29	67.0^a^	5.3^a^	6.3^a^	31.2	38.3^a^

*HD* Heading Date, *PL* Plant Height, *SNP* Spike Number per Plant, *LS* Length of the main Spike, *GNS* Grain Number of the main Spike, *WTG* Weight of 1000 Grains

^a^Indicated that there was significant difference between Edamai No.6 and *whs18* at the level of 0.05

### Effect of stage green-revertible albino on photosynthesis relevant traits of barley

Leaf color variations were also associated with abnormal chlorophyll metabolism. We quantified the contents of chlorophylls Edamai No.6 and *whs18* at different developmental stages to determine whether the albino phenotype of *whs18* resulted from impaired chlorophyll biosynthesis. As expected, the amount of chlorophylls of Edamai No.6 showed no significant change at different stages, while both of chlorophylla and chlorophyllb in *whs18* showed a significant dynamic change of decrease to increase along with the leaf color changing from green to albino to green. The total chlorophyll in ALs of *whs18* was less than one tenth of total chlorophyll in corresponding leaves of WT plants (Table 2).

**Table 2:** Concentration of chlorophylls in Edamai No.6 and *whs18*

ChlorophyIIs (mg/g)		GL	EL	AL	SW	FL
Total chlorophyII	Edamai No.6	1.417 ± 0.063	1.582 ± 0.131	1.340 ± 0.036	1.34 ± 0.036	1.772 ± 0.072
	*whs18*	1.283 ± 0.073^a^	0.828 ± 0.094^b^	0.091 ± 0.009^b^	0.564 ± 0.049^b^	1.533 ± 0.060^a^
ChlorophyIIa	Edamai No.6	1.194 ± 0.053	1.330 ± 0.168	1.071 ± 0.032	1.071 ± 0.032	1.408 ± 0.065
	*whs18*	0.976 ± 0.049^a^	0.701 ± 0.082^b^	0.085 ± 0.007^b^	0.424 ± 0.040^b^	1.257 ± 0.056^a^
ChlorophyIIb	Edamai No.6	0.271 ± 0.009	0.253 ± 0.068	0.268 ± 0.011	0.268 ± 0.011	0.364 ± 0.011
	*whs18*	0.245 ± 0.033	0.127 ± 0.069^b^	0.006 ± 0.003^b^	0.140 ± 0.113^b^	0.324 ± 0.075

*GL* Green Leaf at the seedling stage, *EL* Etiolated Leaf, *AL* Albinistic Leaf, *SW* Stripe White leaf, *FL* Flag Leaf

^a^Indicated that there was significant difference between Edmai No.6 and whs18 at the level of *p* = 0.05

^b^Indicated that there was significant difference between Edmai No.6 and whs18 at the level of *p* = 0.01

**Fig. 3:**
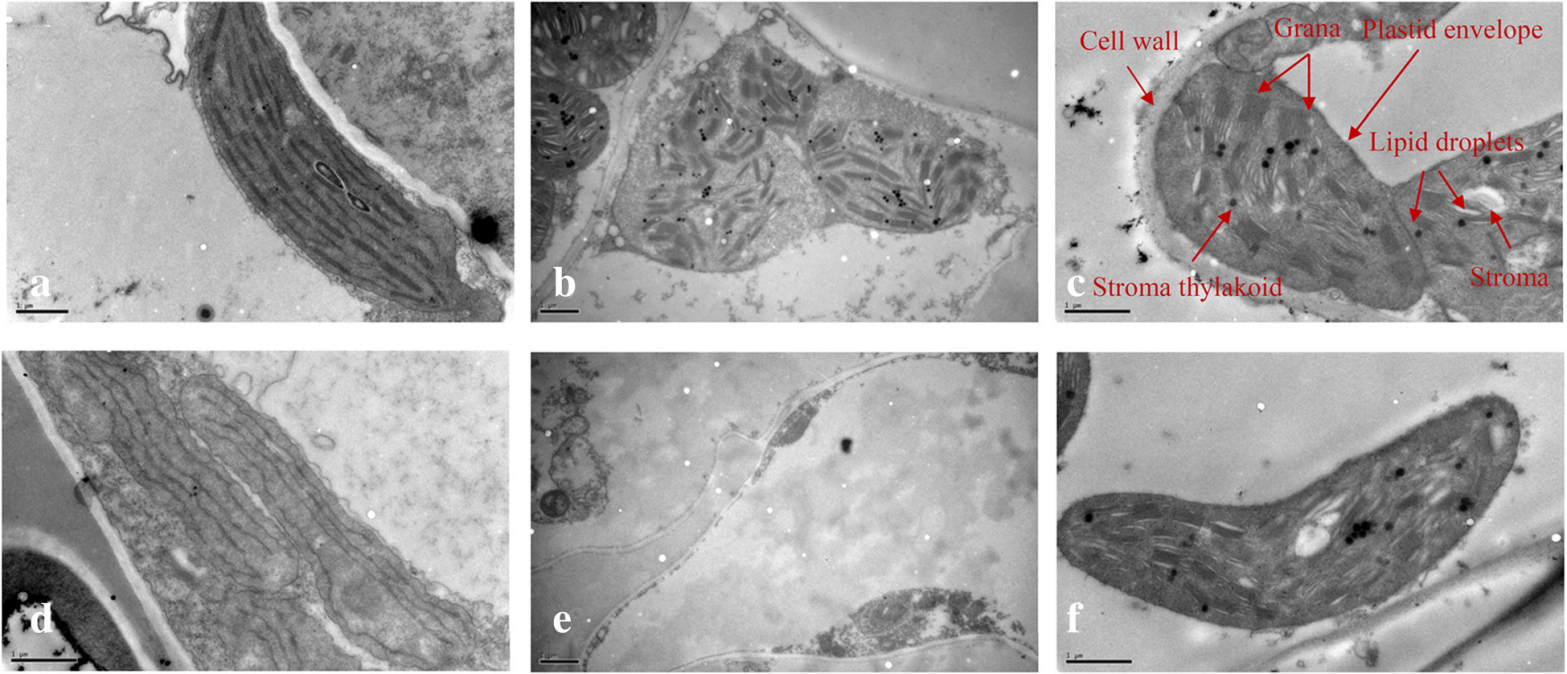
Ultrastructure of chloroplast of Edamai No.6 and *whs18* at different developmental stages. **a** Edamai No.6 at etiolated stage, **b** Edamai No.6 at albino stage, **c** Flag leaf of Edamai No.6 **d** Etiolated *leaf of whs18*, **e** Albinistic leaf of *whs18*, **f** Flag leaf of *whs18*

Ultrastructure of chloroplast in EL, ALand flag leaf (FL, normal green) of Edamai No.6 and *whs18* was also observed at the corresponding stages. As shown in Fig. 3, stacked grana and stroma thylakoid was clearly observed and arranged regularly in Edamai No.6 at all of the three stages (Fig. 3a, b and c). However, in EL of *whs18*, there was no clear boundary between chloroplast and the cell wall. Grana could not be stacked normally in EL of *whs18*, and grana and stroma thylakoid was linear (Fig. 3d) in these ELs. What’s more, ultrastructure of chloroplast was disrupted completely in AL of *whs18* (Fig. 3e). We couldn’t observe any structure of chloroplast in the whole scope. Subsequently, ultrastructure of chloroplast in FL of *whs18* was intact (Fig. 3f) and similar to that of Edamai No.6 (Fig. 3c).

### Genetic analysis of the gene (s) controlling the mutant phenotype

To illustrate the genetic characteristics of the gene (s) controlling the phenotype of stage green-revertible albino, *whs18* was crossed with two wild-type barley cultivars (Table 3). Our analysis showed that all of the four F_1_ population consisted of about 100 individuals showed normal phenotype, either use *whs18* as the male or female parent. For all of the tested F_2_ populations, the segregation ratio of green and albino plants fit a 3:1 Chi square test (Table 3), suggesting that the inheritance of the stage green revertible albino in barley follows a simple Mendelian inheritance pattern and behaves as a single recessive trait, and the gene controlling the trait was designed as *HvSGRA* in this study.

**Table 3:** Genetic analysis of the albino mutant

Cross	No. of normal plant	No. of mutant plant	Expected ratio	Calculated ratio
				*χ* ^2^ (0.05,1) = 3.84
Edamai No.9706 × *whs18*	397	117	3:1	1.37
Edamai No.934 × *whs18*	266	94	3:1	0.24
*whs18* × Edamai No.9706	221	65	3:1	0.79
*whs18* × Edamai No.934	164	51	3:1	0.19

### Preliminary mapping of the *HvSGRA* gene

**Fig. 4:**
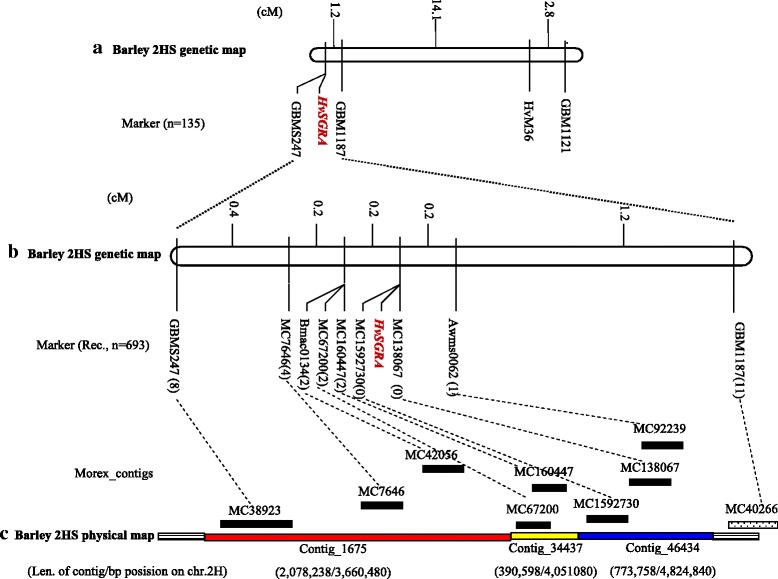
Mapping of *HvSGRA* gene. (*a*) Primary mapping of *HvSGRA* (*b*) Fine mapping of *HvSGRA* (*c*) Physical mapping of *HvSGRA*

To map the *HvSGRA* gene, an F_2_ mapping population was generated from the cross between Edamai No.9706 and *whs18*. More than 400 SSR primer-pairs distributing on the seven barley chromosomes were used to assay the two parents. About one-third of them showed polymorphism between the two parents. Further BSA analysis indicated that the *HvSGRA* gene was on chromosome 2H of barley. Thus SSR markers on 2H were screened in the F_2_ population consisting of 135 albino individuals, and the *HvSGRA* gene was mapped with two SSR markers *GBMS247* and *GBM1187* within a genetic distance of 1.2 cM (Fig. 4a). Further analysis anchored the two markers on morex_contig_38923 and morex_contig_40266 (Fig. 4c), respectively. The two contigs (morex_contig_38923 and morex_contig_40266) were genetically positioned at 2.2622 and 7.4363 cM of barley 2H chromosome, respectively.

SLAF-seq was conducted on the two parents and two bulks with 50 normal or mutant individuals from the F_2_ population, respectively. The SLAF-seq data analysis generated 1.39 Gb, 1.39 Gb, 3.59 Gb and 3.54 Gb data for Edamai No.9706, *whs18*, dominant bulk and recessive bulk, respectively. A total of 100,926 SLAFs were generated, including 10,278 SNPs, 467 repeat, and the other 87,569 showed no polymorphism between the two parents. 59 Diff-markers that are likely associated with the trait were identified after statistic analysis. A total of 38 of them could be anchored to the morex_contigs (Additional file 1). Then five regions with three or more Diff-markers were considered for further analysis, including 2.1955 to 7.4363 cM and 52.4788 to 58.0524 cM on the short arm of chromosome 2H, 49.5042 to 56.1615 cM on the short arm of 6H, 113.2082 to 119.0587 cM on the long arm of 6H, and 104.8159 to 110.2691 cM on the long arm of 7H (Additional file 1). Number of Diff_marker of each region was six, four, five, four and four, respectively (Additional file 1).

### Fine mapping of the *HvSGRA* gene

The result of the preliminary mapping using SSR and SLAF-seq suggested that the *HvSGRA* gene was in the interval of 2.2622 to 7.4363 cM on 2H of barley. In order to further fine map the *HvSGRA* gene, the closest linked SSR marker *GBMS247* and *GBM1187* were used to screen a larger F_2_ population consisting of 693 albinos, and totally 19 recombinants were identified. Two other SSR markers (*Bmac0134* and *Awbms0062*) between *GBMS247* and *GBM1187* were scored on the panel of 19 recombinants for the *HvSGRA* interval. The candidate gene was finally located between *Bmac0134* and *Awbms0062* (Fig. 4b). The two SSR markers were anchored to morex_contig_42056 and morex_contig_92239 (Fig. 4c), respectively.

**Fig. 5:**
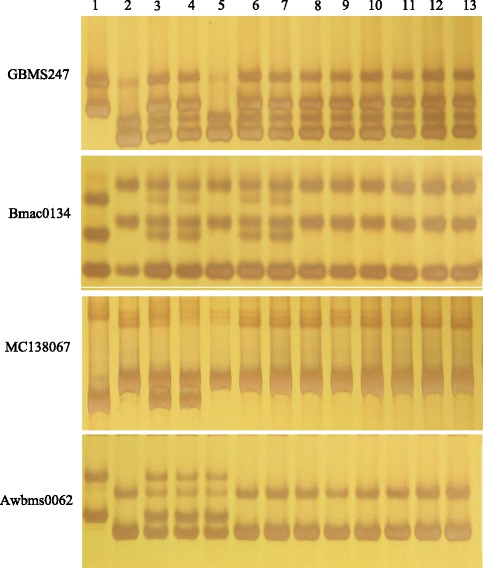
Genotype of the two parents and lines from the derived F_2_ population. Lane1: Edamai No.9706, Lane2:whs18, Lane3-4: Wild-type lines, L5-13: Mutant Lines

For our further analysis, several primer-pairs based on eight morex-contigs nearby morex_contig_42056 and morex_contig_92239 were designed and used to generate new molecular markers potentially linked to *HvSGRA* gene. Five primer-pairs of them showed polymorphism between the two parents (Additional file 2), including MC7646, MC67200, MC160447, MC1592730 and MC138067. As shown in Fig. 5, *HvSGRA* showed cosegregation with MC1592730 and MC138067 in the larger population consisting of 693 albino individuals. Finally, the *HvSGRA* was mapped between morex_contig_160447 and morex_contig_92239, with the genetic distance being 0.2 cM and 0.2 cM (Fig. 4b and c), respectively. They were anchored to two adjacent FP_contigs of barley, contig_34437 and contig_46434 (Fig. 4c), respectively.

### Candidate gene analysis of *HvSGRA*

Our further analysis showed that there were 27 and 33 morex-contigs on contig_34437 and contig_46434, respectively. Six putative genes with high confidence on these morex_contigs were extracted from the most current POPSEQ barley map [34]. They included cytochrome P450, fructokinase-1-like (FLN), Laccase and unknown genes (Table 4).

**Table 4:** Putative genes in the candidate region

Morex_contig	FP_contig	HC_genes_CDS	Gene annotation
morex_contig_160711	contig_34437	AK249799.1	fructokinase-1-like (partial sequence)
morex_contig_244330	contig_34437	AK372445	cytochrome p450 (partial sequence)
morex_contig_67200	contig_34437	MLOC_75134.1	cytochrome p450 (full length)
morex_contig_1592730	contig_46434	AK356172	uncharacterized protein (partial sequence)
morex_contig_6591	contig_46434	AK369630	cbs domain containing protein (partial sequence)
morex_contig_138067	contig_46434	MLOC_6767.1	Laccase (full length)

**Fig. 6:**
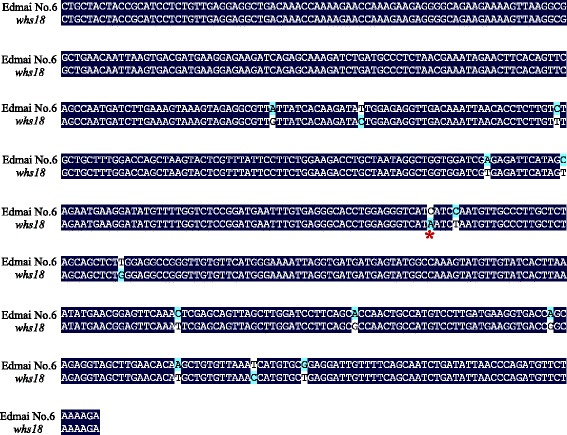
Sequence comparison of the third extron of *HvFLN1* gene between Edamai No.6 and *whs18*. The *red* * indicated the SNP that generated the premature stop codon in *whs18*

**Fig. 7:**
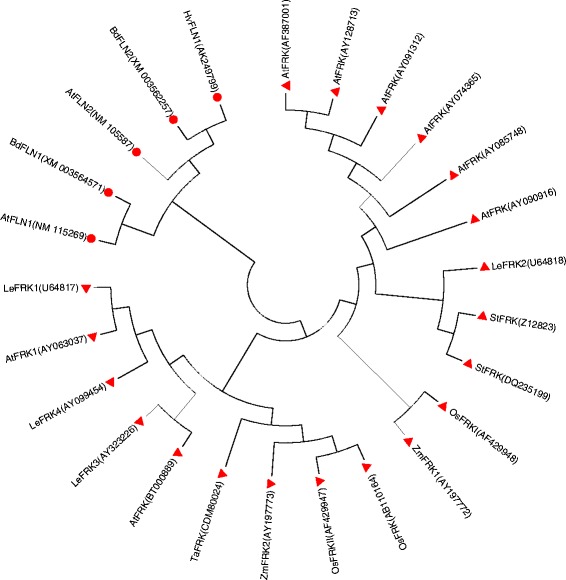
Homology analysis of fructokinase and fructokinase-like proteins in plant. “red circle symbol” indicated the FLNs proteins, “red triangle symbol” indicated the FRKs proteins

All of these six genes were cloned and sequenced from the wild-type Edamai No. 6 and the mutant *whs18*. Our final comparison between them showed that there were totally14 stable SNPs in the third exon of fructokinase-1-like gene *HvFLN1* (Genbank: AK249799) between Edamai No.6 and *whs18* (Fig. 6) both on DNA and cDNA levels. Most importantly, one of these SNPs was a substitution from C to A in *whs18*. The substitution generated a premature stop codon of TAA in *whs18* (Fig. 6). The coding region of the wild-type *HvFLN1* was 1734 bp in length and the substitution lead to the missing of 330 animo acids in C terminal of HvFLN1 in *whs18*. Thus, the mature product of *HvFLN1* in *whs18* was lack of the ATP binding site of the kinase, which may lead to loss function of this gene in *whs18*. The coding regions of the other five genes were exactly the same between Edamai No. 6 and *whs18*. Our further analysis showed that the putative amino acid sequence encoded by *HvFLN1* showed 27 and 41 % similarity to AtFLN1 (Genbank: NP_190977) and AtFLN2 (Genbank: NP_177080) of *Arabidopsis*, respectively (Fig. 7).

## Discussion

Hundreds of mutants with altered leaf color have been previously described in plants, especially in *Arabidopsis* (http://www.arabidopsis.org) and rice (http://rmd.ncpgr.cn/) including green-revertible albino mutants. However, few were described in barley. C*ytoplasmic line 2* (*CL2*) was a cytoplasmically inherited chlorophyII-deficient mutant of barley. At emergence, the *CL2* seedlings-phenotype varied from a grainy light green to an albino color, and gradually greened during the following days [35]. In this study, *whs18*, a natural leaf color mutant of barley cultivar Edamai No.6, was characterized and analyzed. Leaf color of *whs18* was not only different from the wild type, but also was different at different developmental stages (Fig. 1), including GL, EL, ALand SW. This phenomenon was called stage green-revertible albino in rice [9, 13, 17, 19, 36]. There has been no report about this type of leaf color variation in barley. Characterization of the mutant and fine mapping of the gene will pave the way for map-based cloning of the gene and unraveling the molecular mechanisms of the variation. The mutant will also be a new gene resource for barley molecular breeding in future.

Several leaf color variations have been reported to be induced by low temperature, such as the maize inbred line A661I, exposure of whose seedlings to low temperatures during early leaf biogenesis led to chlorophyll losses [15]. Rice mutant *low temperature albino 1* (*Ita 1*) showed albino leaves before 4-leaf stage when grown under temperature lower than 20 °C [10]. Another rice mutant *mr21* showed yellow leaf color under the temperature lower than 25 °C and turned green with an increase in temperature [37]. However, in the barley *Cytoplasmic line 2* mentioned above, higher temperatures during seed formation were negatively associated with pigment content in the seedlings, while higher temperature during the growth of its seedlings had an opposite effect on pigment content [35]. The abnormal leaf color of the rice mutant *hfa-1* seemed to be induced by warm temperature too. Leaf color of *hfa-1* was albino when the temperature was between 25-30 °C, while it was yellow under the temperature of 15-20 °C [36]. To analyze the interaction between the color variation of *whs18* and environment, *whs18* was subjected to three different treatments. As shown in Fig. 2, both of the seedlings in the incubator and plastic membrane showed normal green leaves, while the seedlings grown outside showed abnormal leaf color after one month later (Fig. 2a and b). Furthermore, seedlings sowing at different dates showed etiolation nearly at the same time (Fig. 2c and d), suggesting that the abnormal leaf color of *whs18* was mainly induced by low temperature. Actually, to analyze the precise shift temperature of the leaf color variation in *whs18*, the 10-day-old seedlings of *whs18* have been treated at the temperature of 10/0 °C, 10/4, 15/8 and 20/13 °C (day/night, 12/12 h) for one month, respectively. However, we didn’t observe any color variation under those controlled conditions. On the other hand, the maximum and minimum temperatures were also recorded from mid-November (10-day-old seedling) to the end of December (albino leave emerged) in 2014. We found that the minimum temperature of 10 days was 0 °C and 7 days was lower than 0 °C during this time (Data not shown), suggesting that the temperature under the ice-point may be crucial for the leaf color variation of *whs18*.

As we all known, most of the leaf color variation couldn’t survive under normal condition, especially these albino mutants. Stage green-revertible albino mutant was a special kind of leaf color mutant. This type of mutant could develop normal green leaves after the abnormal ones, which enabled it to flower and mature normally. For example, the green-revertible albino in rice Qiufeng M lead to the significant reduction of neck length and 1000-grain weight at 1 % level, while the other agronomic characters investigated showed no significant difference between the mutant and the wild-type [9]. As compared to Edamai No.6, the abnormal vegetative growth in *whs18* lead to the reduction of plant height and other yield related traits, including SNP, LS, GNS and WTG, and the HD was about eight days later in *whs18* (Table 1). It was probably because that the duration of *whs18* with abnormal leaf color lasted for about three months, which was nearly half of the whole growing season of barley in Wuhan city. The abnormal chlorophyll metabolism in these leaves (Table 2, Fig. 3) influenced the photosynthesis of these leaves, and then played some negative impact on these agronomic traits investigated.

Most of the leaf color variations were controlled by one single recessive nuclear gene, and a few of them were also reported to be controlled by cytogene, such as the stage albinism line of winter wheat FA85 [38]. While the abnormal leaf color of Mt135 was controlled by nucleo-cytoplasmic gene interaction [2]. Our genetic analysis suggested that the stage green-revertible albino of *whs18* was a qualitative trait that controlled by one single recessive nuclear gene (Table 3) , which allowed us to hunt for this gene directly using the strategy of BSA based on molecular markers. We preliminarily mapped the *HvSGRA* gene on chromosome 2H of barley using traditional BSA method.

Recently, a large amount of data from NGS and modern tools enable us to extract putative genes in certain regions without a precise genomic sequence in barley. The strategy of mapping-by-sequencing has accelerated the forward genetics significantly in barley [39]. Using the approach of mapping-by-sequencing applied on the whole-exome capture data, *HvPHYTOCHROME C* was identified as a candidate gene for the early maturity locus modulating the circadian clock and photoperiodic flowering in barley [40]. We assumed to conduct SLAF-seq to develop closer SNP markers linked to the gene. Five regions were identified by SLAF-seq and BSA, including the interval identified by traditional BSA (Additional file 1). Xia et al. [30] also identified three independent candidate regions on chr3 of maize for a qualitative trait using the strategy of BSA based on SLAF-seq, with only three Diff_markers for each region. Actually, SSR markers in the other four regions identified by SLAF-seq (Additional file 1) were also employed in our preliminary mapping analysis. These markers showed no polymorphism either between the two parents or the two pools, and they were excluded from our further analysis. Thus the overlapped region identified by SSR and SLAF-seq was considered the candidate region. On the other hand, marker density (Six Diff_markers) in the *HvSGRA* interval (2.1955 to 7.4363 cM on the short arm of chromosome 2H) identified by SLAF-seq may be insufficient to pin the candidate gene in barley (Additional file 1). Similar phenomenon was also observed in cotton [28]. And our further map-based cloning based on the physical map of barley narrowed down the *HvSGRA* interval to two morex_contigs on two adjacent FP_contigs of barley, contig_46434 and contig_34437 (Fig. 4).

The recent release of the barley draft genome assembly [33] provides a resource for pursuing the *SGRA* gene, however, the precise genomic sequence is still not available. Synteny-based isolation of genes conserved across cereal species is a common and effective way in map-based cloning of barley genes [41, 42]. Whereas, our in silico comparison showed that only 18 of the 60 morex_contigs on FP_contig_46464 and FP_contig_34437 could be anchored to the genome of *Brachypodium distachyon*. Nine and seven of these 18 morex_contigs were distributing on Bd1 and Bd3, respectively (Data not shown). Furthermore, as for the two morex_contigs surrounded the *HvSGRA* gene, morex_contig_160447 was anchored to Bd5 of *Brachypodium distachyon*, and we couldn’t find any homolog contigs for morex_contig_92239. The similar result was also observed in rice. This hindered us from getting a precise physical order of these morex_contigs in the chromosome region containing the *HvSGRA* gene. So all of the genes in FP_contig_46434 and FP_contig_34437 with high confidence were extracted from the POPSEQ barley map [34]. Finally, six genes were identified as candidate gene for *HvSGRA* (Table 4), including four genes with known function, namely cytochrome P450, fructokinase-1-like (FLN1), Laccase. Out of these genes, cytochrome P450 [18] and fructokinase (FRK) [43, 44] have been reported to be associated with albino in plant. Our further analysis showed that one base’s substation of C to A in the third exon of *HvFLNs gene* generated a premature stop codon in *whs18* (Fig. 6a). The lack of ATP binding site of HvFLN1 in *whs18*, which is essential for the kinase activity, may lead to loss of function of this gene. FRK is a kind of kinase that primarily phosphorylates fructose in carbohydrate metabolism, which is involved in many developmental and physiological processes of plant [45]. Except for FRKs, there is still a kind of members called FLNs in plants (Fig. 7). These FLNs were distinctly grouped for a new branch during the homology analysis (Fig. 7), suggesting that they may play different roles in plant as compared to FRKs. However, these FLNs were quite conserved among different species, especially the C terminal of these proteins. The HvFLN1 identified in our study showed high level of similarity to AtFLN1 and AtFLN2 of *Arabidopsis*, respectively (Fig. 7). The most recent investigations showed that the mutation of AtFLN1 or AtFLN2 in *Arabidopsis* displayed a pale-yellow or albino cotyledons phenotype and were seedling lethal on MS medium without sucrose, and only could survive on sucrose-containing medium [43, 44]. The high similarity between the barley gene and its counterparts of *Arabidopsis* suggested that they may play a similar role in developmental and physiological processes of plant. And further functional validation of the candidate gene, such as knock out or functional complementation, was also needed to confirm the role of *HvFLN1* in the green-revertible albino phenotype of barley. Illustrating the molecular mechanisms of this interesting phenomenon will also help us to understand it well, which will be helpful for further use of the gene and the trait in future.

## Conclusions

SSR assay and SLAF-seq in conjunction with BSA were conducted to map a gene controlling the stage green-revertible albino in barley. The *HvSGRA* gene was mapped between two adjacent FP contigs of barley. Substitution of C to A in the third exon of *fructokinase-1-like gene* in *whs18* may be responsible for the stage green-revertible trait of barley. The current study will lay the foundation for hierarchical map-based cloning of the *HvSGRA* gene and utilizing the gene or the trait in the molecular breeding of barley in the future.

## Methods

### Measurement of agronomic traits of *whs18* and Edamai No.6

“*whs18*” was initially isolated from the elite malting barley cultivar Edamai No.6 as a spontaneous mutant in the field. Leaf color of *whs18* showed stage green-revertible albino. Edamai No.6 and *whs18* were planted in the field, and the management of the filed experiments was in accordance with local standard practices. At maturity, agronomic traits including plant height (PL), heading date (HD), spike number per plant (SNP), length of the main spike (LS), grain number of the main spike (GNS) and weight of 1000 grains (WTG) were measured for ten plants of Edamai No.6 and whs18.

### Measurement of concentration of chlorophyll

Concentration of chlorophylls in leaves of *whs18* with different colors were measured [46], and also that in corresponding leaves of Edamai No.6. In brief, healthy and fresh leaves with different color were collected at different stages, namely green leaves (GL) at seedling stage, etiolated leaves (EL) at etiolation stage, albinistic leaves (AL) at albino stage and flag leaves (FL) at heading stage, and cut into sections of 1 mm to 2 mm. Half of one gram of each sample was transferred into a mortar with 25 ml 95 % ethanol and grounded until the leaves turned into white. The tissue was transferred into 50 ml volumetric flask and brought up to volume by 95 % ethanol. The extraction solution was used to measure the absorbance values under 663 nm and 645 nm using a spectrophotometer with 95 % ethanol as control. Each sample was assayed with three biological replicates, and content of chlorophyll was calculated according to the following equations:

$$ \begin{array}{l}{\mathrm{C}}_{\mathrm{T}}={\mathrm{C}}_{\mathrm{a}}+{\mathrm{C}}_{\mathrm{b}}\\ {}{\mathrm{C}}_{\mathrm{a}}=\left(12.7{\mathrm{A}}_{663}-2.59{\mathrm{A}}_{645}\right) \times \mathrm{v}/\left(1000\times \mathrm{m}\right)\\ {}{\mathrm{C}}_{\mathrm{b}}=\left(22.9{\mathrm{A}}_{645}-4.67{\mathrm{A}}_{663}\right) \times \mathrm{v}/\left(1000\times \mathrm{m}\right)\end{array} $$

C_T_ was the content of total chlorophyll with unit of mg/g fresh weight. A_663_ and A_645_ were the absorbance at 663 nm and 645 nm, respectively. V is the final volume of the extraction solution (mL), and m is the initial weight of leaves (g).

### Transmission electron microscopy (TEM) assay

Corresponding leaves of Edamai No.6 and *whs18* at three of the five developmental stages were sampled for TEM assay [10], including etiolated leaves, albinistic leaves and flag leaves. For detailed, leaves were cut into sections of 1 mm × 1 mm and fixed in 2.5 % glutaraldehyde in 0.1 mol/L phosphate buffer (PBS, pH 7.2) at 4 °C for 3 days, following by washing with 1 mol/L PBS for three times, 15 min per time. Samples were fixed in 1 % osmium tetroxide in 0.1 mol/L PBS for about 2.5 h until it turned into black, followed by washing with 1 mol/L PBS as before, then dehydrated with a gradient acetone series (30-50 %-70-80 %-90-100 %), 30 min for each concentration, and embedded in Polybed 812 (Epon) resin. Ultrathin sections were obtained with an ultramicrotome, mounted on grids, stained by lead citrate and uranyl acetate for 10 and 30 min, respectively. The final dried ultrathin sections were observed and photographed using a transmission electron microscope H-7650 (Hitachi, Japan) according to the protocol provided by the manufacture.

### Temperature treatment

To characterize whether the stage green-revertible albino of *whs18* was induced by low temperature, the 10-day-old seedlings of *whs18* grown in pots were transferred into the incubator with 18/10°Cday-night temperature (T_2_), or 10-day-old seedlings of *whs18* grown in the field were covered with two layers of plastic membrane (T_4_). Leaf color of them was compared with the corresponding seedlings grown under the normal condition, which were referred to T_1_ and T_3_ for the pots and the field, respectively. *whs18* was also sowed at three different dates with 10 days’ interval in 2012 and 2013, including normal sowing date (Nov.7^th^) and 10 days’ earlier (Oct.28^th^) or later than (Nov.17^th^) the normal sowing date, and the leaf color was also observed at different stages.

### Genetic analysis of the gene controlling the stage green-revertible albino of barley

Genetic characterization of the gene (s) controlling the stage green-revertible albino of *whs18* was analyzed based on the phenotype of F_1_ hybrid and F_2_ population derived from four crosses, including Edamai No.9706 × *whs18*, Edamai No.934 × *whs18*, *whs18* × Edamai No.9706 and *whs18* × Edamai No.934. Chi-squared test was used to determine the suitability of observed data with expected segregation ratios.

### SSR-based genotyping and bulked segregant analysis

To determine the position of the stage green-revertible albino gene *sgra* in “*whs18*” on genetic map, an F_2_ population derived from barley cultivar Edamai No.9706 and *whs18* was used. Traditional BSA strategy was conducted to prepare genomic DNA pools for analysis. To be detailed, genomic DNA of 10 green lines and 10 albino lines from the F_2_ population along with the two parents was extracted using the modified CTAB method [47]. Equal amounts of DNA from the 10 green or albino lines were pooled together to construct the dominant or recessive bulks, respectively. More than 400 known SSR markers from GrainGenes (http://wheat.pw.usda.gov/GG2/index.shtml) distributing on the seven chromosomes of barley were screened in the parents and the two bulks. PCR reaction and the polyacrylamide gel analysis were conducted to analyze the genotype of the samples according to Liu et al. [48]. The probably polymorphic markers that associated with the trait were used to genotype the 135 homozygous recessive individuals from the F_2_ population firstly. Linkage between molecular markers and the *SGRA* gene was analyzed using Mapmaker 3.0b [49] with an LOD score of 3.0 as the threshold. The genetic map was drawn with the software Mapdraw V2.1 [50]. Closest linked markers were used as queries to search in the database of assembly_WGS Morex on IBSC [33] using BLASTN and were anchored to the genome of barley.

### SNP-based genotyping and bulked segregant analysis

To narrow down the candidate gene interval, SLAF-seq [22] was conducted with Illumina Genome Analyzer II (Illumina Inc., San Diego, CA, USA) to develop SNP markers that probably linked to the gene. Specifically, genomic DNA of 50 normal green and 50 albino plants from the F_2_ population along with the two parents was extracted as mentioned above. Equal amounts of DNA from the 50 normal green or albino plants were pooled together to construct the dominant or recessive bulks, respectively. The totally four samples were subjected to SLAF-seq and SNP-index analysis. The procedure was performed as described by Sun et al. [22]. Data analysis and association analysis was the same as Xia et al. and Chen et al. [28, 30]. Diff-markers likely associated with the trait were anchored to the genome of Morex (Data version: assembly3_WGSMorex) [33] using blat [51]. The chromosome interval with five or more consecutive Diff-markers was considered for further analysis.

### Fine mapping of the *HvSGRA* gene

The closest markers linked to the candidate gene were screened in a larger F_2_ population consisting of 693 homozygous recessive individuals derived from Edamai No. 9706 and *whs18*. More SSR markers between or nearby the closest markers were genotyped in the resulting recombinants to narrow down the interval containing the candidate gene, which had been marker-selected from the larger population. The markers were anchored to morex_contigs as mentioned above. Morex-contigs nearby them were picked up and used to generate new molecular markers potentially linked to *HvSGRA* gene. Primers were designed using the DNAMAN software.

### Physical mapping of the *HvSGRA* gene and candidate gene analysis

The closest markers linked to the candidate gene were anchored to the barley physical map by doing blast with BAC end sequences and fully sequenced BACs [33], and the putative FingerPrinted contigs (FP contig version: fpc_10) containing *HvSGRA* gene were identified. Then morex contigs on the FP contigs were extracted from IBSC, and putative genes with high confidence on these morex_contigs were extracted from the most current POPSEQ barley map [34]. Genes were annotated after running blastn search against the database of HC_genes_CDS_Seq of barley [33].

As shown in Table 4, some morex_contigs only contained partial sequence of the genes, so the full genomic sequence of these genes were first cloned in silico. Several overlapped primers were designed based on the sequence of the Morex-contigs and the candidate genes using DNAMAN5.0 software. The specific PCR product from DNA and cDNA of the wild-type Edamai No.6 and the mutant *whs18* was sequenced and compared.

Amino acid sequences of FRKs or FLNs from other plant species were downloaded from NCBI (www.ncbi.nlm.nih.gov). The deduced amino acid sequence of *HvFLN* and FRKs or FLNs from other plant species were aligned and compared using the software MEGA 5.1.

## Additional files

Additional file 1:
**Comparison between the markers identified by SLAF-seq and SSR assay.** (XLSX 13 kb) Additional file 2:
**Information of the HvSGRA associated markers.** (XLSX 10 kb)

## Abbreviations

*SGRA*: Stage Green-Revertible Albino Gene
BSA: Bulked Segregant Analysis
SLAF-seq: Specific Length Amplified Fragment Sequencing
GL: Green leaf at the seedling stage
EL: Etiolated leaf
AL: Albinistic leaf
SW: Stripe White leaf
FL: Flag leaf
PL: Plant height
HD: Heading date
SNP: Spike number per plant
LS: Length of the main spike
GNS: Grain number of the main spike
WTG: Weight of one thousand grains
NGS: Next Generation Sequencing
FRK: Fructokinase
FLN: Fructokinase-like protein
